# Opportunities and Barriers to Pediatric Antimicrobial Stewardship by Community Pharmacists

**DOI:** 10.1093/jpids/piae039

**Published:** 2024-04-30

**Authors:** Shahd Alzard, Jane Wen, Nguyen Phuong Quynh Huynh, Shahrzad Shirkhanzadeh, Jocelyn Y Tso, Meynard Rabino, Marijana Vanevski, Penelope A Bryant, Jim Buttery, Gabrielle M Haeusler, Angelina S Lim

**Affiliations:** Pharmacy and Pharmaceutical Sciences Education Department, Faculty of Pharmacy and Pharmaceutical Sciences, Monash University, Melbourne, Victoria, Australia; Pharmacy and Pharmaceutical Sciences Education Department, Faculty of Pharmacy and Pharmaceutical Sciences, Monash University, Melbourne, Victoria, Australia; Pharmacy and Pharmaceutical Sciences Education Department, Faculty of Pharmacy and Pharmaceutical Sciences, Monash University, Melbourne, Victoria, Australia; Pharmacy and Pharmaceutical Sciences Education Department, Faculty of Pharmacy and Pharmaceutical Sciences, Monash University, Melbourne, Victoria, Australia; Pharmacy and Pharmaceutical Sciences Education Department, Faculty of Pharmacy and Pharmaceutical Sciences, Monash University, Melbourne, Victoria, Australia; Pharmacy and Pharmaceutical Sciences Education Department, Faculty of Pharmacy and Pharmaceutical Sciences, Monash University, Melbourne, Victoria, Australia; Pediatric Infectious Diseases Unit, Royal Children’s Hospital, Melbourne, Australia; Pediatric Infectious Diseases Unit, Royal Children’s Hospital, Melbourne, Australia; Department of Paediatrics, University of Melbourne, Melbourne, Australia; Pediatric Infectious Diseases Unit, Royal Children’s Hospital, Melbourne, Australia; Department of Paediatrics, University of Melbourne, Melbourne, Australia; Pediatric Infectious Diseases Unit, Royal Children’s Hospital, Melbourne, Australia; Department of Paediatrics, University of Melbourne, Melbourne, Australia; Pharmacy and Pharmaceutical Sciences Education Department, Faculty of Pharmacy and Pharmaceutical Sciences, Monash University, Melbourne, Victoria, Australia; Hormone Research, Murdoch Children’s Research Institute, Melbourne, Australia

**Keywords:** AMS, antibiotics, community pharmacists, parents, pediatric, role

## Abstract

Community Pharmacists (CPs) are easily accessible and can advocate for the appropriate use of antibiotics in children. Semi-structured interviews were conducted with 47 CPs and 46 parents/caregivers. Both groups expressed challenges to intervening when antibiotics have already been prescribed and highlighted the need for more support for CPs to make informed decisions.

## INTRODUCTION

More than 3 million antibiotic prescriptions were dispensed in Australia for children aged 9 years and under in 2016–2017, nearly 1 antibiotic course per child per year [1]. Unnecessary and inappropriate antimicrobial prescribing for self-limited childhood illnesses is a global concern [2]. Various factors might contribute to this issue including parental health knowledge, expectation for antibiotics, unwarranted childcare policies, and pressure on prescribers to maintain good rapport with families [3, 4]. Whilst pharmacist-led Antimicrobial stewardship (AS) interventions have been well received in the hospital setting [5], community pharmacists (CPs) are an underutilized AS resource [6, 7]. Because CPs are generally the first source of medical information for parents and interact with general practitioners (GPs), also known as family physicians regularly, they are well-positioned to advocate for AS in children [6, 7]. CPs can utilize their knowledge to help differentiate between viral and bacterial causes when encountering initial phases of infections, recommend evidence-based, and safe symptomatic interventions when antibiotics are not indicated, and refer to GPs when appropriate [7–10]. Implementation of AS in the community could benefit public health and increase job satisfaction by allowing CPs to take on more responsibility and further professional development [8]. Perceptions on CPs role in AS has not been explored widely, especially not from the perception of patients and their caregivers. Our research aims to address this gap, by exploring CPs’ and parents’ experiences, opinions, and knowledge regarding antibiotic use in children and the role of CPs in advocating for AS.

## METHODS

CP and parent/caregivers of children under 16 were invited to participate in semi-structured interviews (Supplementary Appendix 1) by flyers/email which were sent to various community pharmacies and early learning centers across Victoria. Parents/caregivers were excluded if they or their partner currently worked or have worked as a pharmacist (in any setting). CPs who are provisionally or currently registered in Melbourne, Australia and work at least one regular shift per month in a community pharmacy were included. No more than two CPs practicing from the same pharmacy were included, as pharmacists at the same pharmacy are likely to have experienced similar pediatric AS trends. The primary outcomes were the experiences of pediatric antibiotic use in community pharmacies from the perceptions of CPs and parents/caregiver. Data from interview transcripts was analyzed using an inductive and deductive approach and guided by a thematic analysis approach by Braun and Clarke 2006 [11]. This project was approved by the institution’s human research ethics committee.

## RESULTS

Overall, out of 102 eligible participants, 47 interviews with practicing CPs and 46 interviews with parents/caregivers were conducted. The demographics of the participants are shown in Table 1. Five main themes arose from the interviews and data from both groups of participants could be combined across themes. A summary of themes and exemplar quotes can also be found in Table 2.

**Table 1.** Demographics of Participants (CPs)

Demographic	CP (*N* = 47) *n* (%)	Parents/Caregivers (*N* = 46) *n* (%)
Gender		
Female	33 (70.2)	39 (84.8)
Male	14 (29.8)	7 (15.2)
Years worked as a CP		
Intern (provisionally registered CP)	6 (12.8)	–
0–5 y	14 (29.8)	–
5–10 y	17 (36.2)	–
10+ y	10 (21.3)	–
No. of children		
Parent/caregiver of only one child	–	18 (39.1)
Parent/caregiver of two children	–	23 (50.0)
Parent/caregiver of three or more children	–	5 (10.9)
Age of children^a^		
Parent/caregiver of children <5 years old	–	18 (39.1)
Parent/caregiver of children 5–10 years old	–	18 (39.1)
Parent/caregiver of children >10 to <16 years old	–	10 (21.7)
Healthcare worker		
Healthcare worker parent/caregiver	–	19 (41.3)

^a^If more than one child and all children do not fit into a category, base category on oldest child.

**Table 2.** Summary of Themes and Subthemes of CP’s Role in AMS

Theme	Explanation/Commentary	Exemplar Quote
AMS interventions are too challenging for the CP; the perception from both the public and CPs that they can only play a limited role in AMS	CP role in AMS is limited to checking doses, allergies, and managing side effects due to lack of resources and perception from parents, and CPs themselves	“I think I’ve come to expect that healthcare quality is not the same as it used to be. Because people always rush, sometimes people are not fully present when they’re listening.” (Parent 3) “….the pharmacist role is the last point of care between the doctors. I mean the doctor has all patients’ details but sometimes they can miss something. So it’s that additional diligence. So I think pharmacists do have a role” (Parent 3) “Patients’ parents/caregivers might be a little bit hesitant about us calling the doctor, because that means they have to wait” (Pharmacist 29) “….[the parents] went to their GP, they trusted their GP and got educated by doctors, hence I think it is not necessarily asking what it is for” (Pharmacist 8) “I think we play an important role. We can speak to patients about resistance, educate patients in the community about common colds/viral infections/minor illnesses and encourage symptomatic treatments instead of seeking antibiotics. We take an important part in educating patients because doctors may not spend enough time with them” (Pharmacist 8) “….parents are usually understanding when it comes to dosage. But when it comes to me saying ‘it’s inappropriate’ they are less likely to listen.” (Pharmacist 28) “….doctors are not receptive to my suggestions and refuse to change the prescribed antibiotic” (Pharmacist 27) “…want to clarify the dose and strength with the doctor, they might feel offended” (Pharmacist 38) “…because I don’t have any evidence to back myself up like the sensitivity reports. All my judgment is based on what the patient tells me and the guidelines” (Pharmacist 38) “…it is also hard to intervene because we don’t have access to patients’ health records such as microbiological culture, in contrast to hospital settings” (Pharmacist 44) “…more tutorials and lectures are needed in uni on children’s antibiotics. There is vague information about children, mostly adults. For example, it would be useful to know common infections in children and the available treatments (dosage, duration, etc.)” (Pharmacist 6) “I would expect the GP to prescribe it, then I would get the extra information, for example, potential side effects or whether it should be taken with or without food, where it should be kept refrigerated; all that kind of information from the pharmacist” (Parent 6) “I have sought [advice]. Sometimes I just ask for their opinions about whether it’s necessary for my child to take antibiotics or not” (Parent 2)
The main concern is the supply of antibiotics rather than the indication of antibiotics	Pharmacists are more concerned about whether the antibiotic is available or not rather than checking whether the antibiotic is needed or not	“I think that the current problem is shortage” (Pharmacist 34) “I think the big one recently is the shortage of antimicrobials” (Pharmacist 35) “I think generally they have just asked ‘have you used it before?’, and if it’s for yourself or someone else. I don’t know if I’ve been asked what the actual reason I was taking it was.” (Parent 6) “They asked me if I had taken it before, but rarely what it’s for…then they normally check if they have it in stock.” (Parent 7)
A prior successful intervention from a CP helps build rapport and can give the impression that the CP can take on a larger role in AMS	Parents are more receptive to AMS intervention by CPs if there are prior positive experiences	“Parents are usually receptive, listening and thankful that the pharmacist has intervened if the script is inappropriate.” (Pharmacist 27) “Parents realize that we actually care about them, hence they trust us, and become more receptive to the information and reassurance from us.” (Pharmacist 8) “…the pharmacist gave me a really lengthy answer which is very informative, even a better answer than from the GP’s. So I guess this can be classified as a good experience” (Parent 8)
The long-term effects of AMR are not an immediate concern	CPs believe that AMS education is too overwhelming to be included in community pharmacies	“…briefly, finishing the course mainly, but not much about the resistance itself because I don’t want to overwhelm the parents” (Pharmacist 39) “I do bring up to patients to take the antibiotics the number of days that it needs to be used; that’s actually something else that’s lacking from scripts quite often. I think telling the patient antibiotic resistance goes a little bit beyond, they’ve already got enough information” (Pharmacist 40) “What’s the harm in one or two courses of unnecessary standard antibiotics” (Parent 30) “They’re just run of the mill antibiotics, not the hard-core stuff you put in the veins” (Parent 42)
Pharmacists are concerned about AMR but believe accountability currently lies with the prescriber	CPs are aware of AMS and AMR though believing that accountability currently is with the prescribers as they have limited access to information about the patient and their infection	“…resistance is a serious thing…it’s hard to help once the prescriber has made the call to treat with antibiotics” (Pharmacist 27) “I’ve read a lot about it [AMR], it is a real problem, but appropriate prescribing from the prescriber is the key to reducing the problem” (Pharmacist 28) “…it’s just a repeated pattern of antibiotics being prescribed when they are not needed or at wrong dosages” (Pharmacist 44)

*Theme 1: AS interventions are too challenging for the CPs; perception from both parents/caregivers and CPs are that CPs can only play a limited role in AS* found both parents and CPs perceived a limited role for AS due to lack of time and resources. The increased workload for CPs has been noticed by parents as well as CPs, noting that it is difficult for CPs to take on more responsibility. Parents also commented that they have limited time to listen to the CPs after they have already spoken to the doctor about the same issue, and noted they are wary of time pressure that might limit CPs from making interventions. Another barrier to conducting AS by CPs identified by both groups was the perception of healthcare hierarchy. For example, many parents/caregivers believed AS responsibilities lie with the prescriber, while CPs felt their roles were limited to AS interventions such as checking doses and allergies. Additionally, CPs believed that they do not have enough information (eg, microbiology), to make informed decisions. CPs also voiced a desire for more education to build confidence to make more AS interventions, especially for children.

However, some CPs believe that they can play an important, albeit limited, role in managing antibiotic use in the community, including community education on antimicrobial resistance (AMR), differentiation between viral and bacterial infections, symptomatic treatment recommendations, and antibiotic side effect management. This sentiment was also echoed by parents/caregivers, who identified the CP as the last point of care.

*Theme 2: The main concern is the supply of antibiotics rather than the indication of antibiotics* outlines how CPs see their role as mainly supplying the medications, rather than checking indications and the need for antibiotics. This was further highlighted by parents who stated CPs rarely ask for the indication of the antibiotic when presented with a prescription.

*Theme 3: A prior successful intervention from CPs helps build rapport and can give an impression that CPs can take on a larger role in AS* highlighted that parents/caregivers who have pre-established rapport with CPs based on previous experiences are generally receptive to AS interventions. They entrust CPs for clear instruction, duration, and information such as excipients or even flavors of the antibiotic oral suspensions to ensure adherence to the medication. CPs claimed that parents are mostly reasonable, grateful, and were willing to cooperate with CPs to ensure best practice.

*Theme 4: The long-term effects of AMR are not an immediate concern* suggested that most CPs claimed that they would not counsel on the importance of AMR and AS because this would be too overwhelming for parents, instead stating that their counseling would focus on how to take the antibiotic and side effects. Long-term effects also did not appear to be a concern for parents as some believe that one or two courses of unnecessary antibiotics won’t cause harm or believe AMR is more of a concern with intravenous antibiotics.

*Theme 5: Pharmacists are concerned about AMR but currently believe accountability lies with the prescriber* demonstrated that, although CPs are concerned about AMR, they believe that the main contribution to AMR was inappropriate prescribing. This generally includes both unnecessary antibiotic prescriptions for self-limiting conditions, as well as incorrect choice and doses for conditions for which antibiotics are indicated. CPs stating until they can access more information about the patient and the infection (as mentioned in theme 1), their role is limited to checking the safety of doses and allergy contraindications.

## DISCUSSION

Overall, semi-structured interviews with CPs and parents/caregivers of children identified both the potential opportunities for and obstacles to AS in community pharmacy. CP respondents felt AS is important, but that limited resources impacted their confidence and comfortability to implement AS interventions, especially in pediatrics. Parents/caregivers perceive that AS is traditionally the responsibility of prescribers and that it would be challenging for CPs to intervene after a parent/caregiver has already had an assessment with their GP. These data can inform future efforts to implement AS in community pharmacy.

Our results suggest CPs might be considered either as a first or last point of care, which dictates how they might conduct AS. For example, as a first point of contact, CPs could educate parents on the signs and symptoms of viral infections as an opportunity to prevent unnecessary trips to the GP. Alternatively, once a prescription has been given, it would be challenging to modify the appropriate choice of antibiotic based on the indication. Thus, AS opportunities at the last point of care might include de-labeling of “no or low risk” penicillin allergies in children, helping facilitate clinician-directed watch, and wait strategies in common pediatric conditions such as otitis media [7], helping provide anticipatory guidance for recognizing signs and symptoms of adverse drug effects from antibiotic use, conducting home medicines reviews to reduce stockpiling of antibiotics, or delivering education campaigns in the pharmacy to create a greater awareness of AMR.

Like other studies [9, 12], this study also highlighted challenges for CPs including access to microbiology reports (either through electronic health records or in collaboration with pathology centers) as well as finding more accessible communication pathways/channels between the GP and CP to discuss questions regarding antibiotic prescriptions. Having these resources could improve CP comfortability in making AS interventions while supporting future pharmacist-led AS programs that have been successfully achieved in the hospital setting [5].

This study has several limitations. Observations might not generalize beyond the participants sampled or the region of Australia under study. Parent/caregiver responses might have been influenced by the timing and frequency of their child’s antibiotic use, which was not accounted for in the analyses. Lastly, qualitative reports of parent/caregivers and CPs might not reflect true practice. Further research should expand these data and test potential AS interventions in the community pharmacy setting.

## Supplementary Material

piae039_suppl_Supplementary_Appendix

## Notes

***Acknowledgments.*** The research team would like to thank all the participants who took the time to be interviewed for this project.

***Financial support.*** No funding was received by any of the authors for the performance of the work reported in this manuscript

***Potential conflicts of interest.*** My co-authors and I do not have any conflicts of interest to disclose.
