# Supplementary material for: Opportunities and Barriers to Pediatric Antimicrobial Stewardship by Community Pharmacists
Source: J Pediatric Infect Dis Soc. 2024 Apr 30;13(6):313–6. doi: 10.1093/jpids/piae039 (PMC11212362; doi:10.1093/jpids/piae039)
Supplement: piae039_suppl_Supplementary_Appendix [file piae039_suppl_supplementary_appendix.docx]

**Appendix 1:**

**Semi-structured interview guide for community pharmacist interviews:**

***Demographics***

How years have you worked in community pharmacy?

Gender?

***Interview Prompts***

What are the common paediatric antibiotic problems you encounter in community pharmacy?

What do you know about Antimicrobial resistance and antimicrobial stewardship (AMS)? *Further prompt: Did you mainly come to know about AMS whilst practising as a pharmacist, or early on as a student during your undergraduate pharmacy degree?*

What role do you think there is for community pharmacists in antimicrobial stewardship?

Do you practice AMS in community pharmacy, please explain some barriers and facilitators that impact your practice?

When presented with a script for an antimicrobial for a paediatric patient, what questions do you normally ask the parent/carer?

*Further prompt: Do you check with the parent/carer what the antimicrobial is for? If the child has allergies? Microbiology?*

Tell us about any experiences where you have seen antimicrobial inappropriately prescribed for a paediatric patient and did you intervene? If you did intervene, what was the outcome, if you didn’t intervene, what were the barriers stopping you from doing so?

*prompt with (if they have nothing to say) - do you notice antibiotics being prescribed for viral infections? Do you notice incorrect allergy labels? Have you encountered inappropriate antibiotic dosing? If so, tell us about that experience and what you did to resolve the problem?*

Please elaborate on your answer if possible e.g. if you find a lot of antibiotics prescribed for viral infections

When dispensing antimicrobials for paediatric patients, do you ever counsel on the importance of AMS and antimicrobial resistance? Please explain why or why not.

**Semi-structured interview guide for parent/carer:**

***Demographics***

What is your gender?

Please specific the age in years and sex of all your children (or children you care for)

| Child & gender | Age | Sex |
| --- | --- | --- |
| E.g. child 1 |  |  |
| E.g. Child 2 |  |  |

What is your relationship to your children? E.g. mum, dad, grandad

What is your profession?

***Interview Prompts***

Tell me about your experiences where your children have used antibiotics

*Further prompt: What was the antibiotic for, who gave you advice to take the antibiotics? What information you were given about the antibiotics and from whom?*

Tell me what you know about the term ‘antibiotic resistance’

*Prompt: If asked, explain the term to them simply e.g. Antibiotic resistance is the ability of bacteria to resist the effects of antibiotics – that is, the germs are not killed, and their growth is not stopped. Infections with antibiotic resistant bacteria are difficult to treat.*

Please explain the times you have sought advice from your pharmacist in regards to antibiotic use either for yourself or the children you care for?

In your experience, have you found pharmacists always check what the antibiotic is used for?

Has a pharmacist ever spoken to you about antimicrobial resistance? If yes, please detail what was said in the conversation

What roles do you think a pharmacist could have in regards to managing antibiotic use in the community?

What positive experiences (if any) have you had in regards to your experience with community pharmacists and antibiotic use?

*Further prompt: What about other medications?*

What negative experiences (if any) have you had in regards to community pharmacists and antibiotic use?

*Further prompt: What about other medications?*
